# First-day intracranial pressure correlates with ICU mortality in subarachnoid hemorrhage patients: an analysis of the MIMIC-IV database

**DOI:** 10.3389/fneur.2025.1594680

**Published:** 2025-07-02

**Authors:** Zhao Hao, Zhang Ziqian, Cao Hang, Xin Qu, Ning Wang

**Affiliations:** Department of Neurosurgery, Xuanwu Hospital, Capital Medical University, Beijing, China

**Keywords:** subarachnoid hemorrhage, intracranial pressure, intensive care unit mortality, time-series clustering, MIMIC-IV database

## Abstract

**Background:**

The purpose of this study was to explore the optimal intracranial pressure (ICP) distribution of subarachnoid hemorrhage (SAH) patients on the first day in the intensive care unit (ICU) using data mining methods.

**Methods:**

Continuous ICP monitoring records of 176 SAH patients on the first ICU Day were collected from the MIMIC-IV database, comprising individuals treated at Beth Israel Deaconess Medical Center (Boston, MA) ICU between 2008 and 2019. Data underwent five-step processing: rounding (to hour point), missing value imputation, resampling, min-max normalization, and time-series clustering. Four unique clusters of the SAH cohort were identified, differing by daily average and variance of ICP. Propensity score estimation was used to determine the average treatment effect of ICP management on ICU mortality based on the X-tile recommended cut-off point.

**Results:**

The study cohort comprised 176 patients (mean age 58.8 ± 14.54 years; 58.0% [102/176] female) who met the inclusion criteria. The daily average was the only statistically significant factor in the propensity score estimation. A daily average ICP of 14 mmHg was identified as the cut-off point. The group with a daily average ICP above or below the cut-off point was an independent ICU mortality predictor in the multivariate analysis, with the largest odds ratio value among included variables. Notably, the daily average ICP was higher in ICU deaths than in survived patients under similar first-day fluid balance (ICU deaths vs. survived patients: P_Pearson_ = 0.002, *R*^2^ = 0.25; P_Pearson_ = 0.016, *R*^2^ = 0.04).

**Conclusion:**

In the study cohort collected from MIMIC-IV SAH patients, using 14 mmHg as the cut-off point for the daily average ICP on the first ICU Day demonstrated favorable ICU mortality outcomes.

## Background

Severe subarachnoid hemorrhage (SAH) is a significant challenge in intensive care unit (ICU) settings due to its high short-term mortality rate, accounting for approximately 30 to 50% of cases (1, 2). Intracranial hypertension, a common complication of SAH, can exacerbate brain injury in critically ill patients (3). Therefore, monitoring intracranial pressure (ICP) is crucial for assessing intracranial hypertension and providing timely intervention. A delayed diagnosis of intracranial hypertension may lead to severe consequences, including brain herniation, brainstem compression, brain parenchyma and vessel distortion, and ultimately, death (4). Existing literature on ICP values associated with increased risk of death remains inconclusive and conflicting, with various cut-off values proposed (5). One possible reason for this lack of consensus is that previous studies have primarily focused on daily ICP values, neglecting the potential impact of hourly changes. Considering that many SAH patients exhibit variable ICP values within the first day of admission, it is vital to understand the correlation between hourly ICP changes and patient outcomes.

This study aims to address these gaps by utilizing the MIMIC-IV database to cluster SAH patients into four groups based on their ICP values on the first day after SAH. The primary objectives are to explore the hourly variability of ICP and investigate the association between ICP values and mortality in SAH patients. Through this approach, the study seeks to provide a more comprehensive understanding of the role of ICP monitoring in SAH patient management and contribute to the development of more effective treatment strategies.

## Methods

### Study cohort

**Figure 1:**
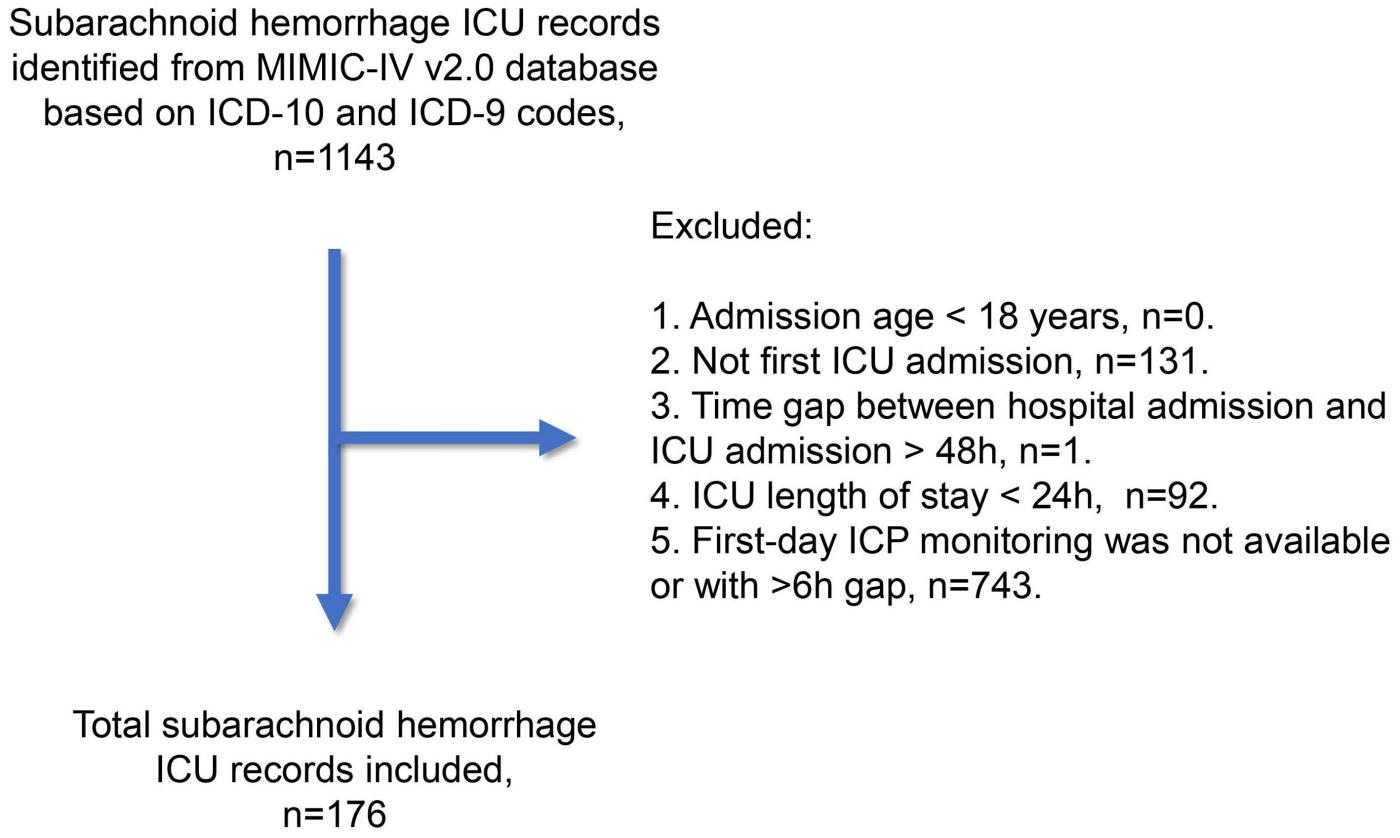
Flow of study cohort establishment in the SAH study.

The subarachnoid hemorrhage cohort in this study was derived from the Medical Information Mart for Intensive Care IV (MIMIC-IV) database (v2.0, available at https://physionet.org/content/mimiciv/2.0/) (6). Patients were treated between 2008 and 2019 in intensive care units at the Beth Israel Deaconess Medical Center in Boston, US. All patient identifiers were removed, and the data was anonymized in accordance with the Health Insurance Portability and Accountability Act safe harbor provision. The first author (HC) completed the CITI Data or Specimens Only Research Training (Certification number: 49999713) required for MIMIC-IV database access and was responsible for data acquisition and primary analysis in this study. As summarized in Figure 1, we utilized ICD-9 and ICD-10 codes to include subarachnoid hemorrhage records from the MIMIC-IV database. The exclusion criteria were as follows: (1) admission age below 18 years; (2) record not pertaining to the first ICU admission; (3) time gap between hospital and ICU admission exceeding 48 h; (4) ICU stay lasting less than 24 h; and (5) unavailability of ICP monitoring during the first ICU Day, or a monitoring gap of over 6 hours. Our study adhered to the Reporting of Studies Conducted using Observational Routinely Collected Health Data statement. The Xuanwu Hospital ethics committee approved the study protocol. The Institutional Review Board at Beth Israel Deaconess Medical Center reviewed the collection of patient information and creation of the research resource, granting a waiver of informed consent and approving the data-sharing initiative.

### Data acquisition and processing

The first-day intracranial pressure (ICP) value at a representative time point was extracted from the MIMIC-IV v2.0 database. As monitoring frequency varied individually, each ICP value’s time point was rounded to the nearest hour. The mean was used for multiple ICP values with the same time point after rounding. We employed the R package imputeTS (version 3.3) to impute missing ICP values using the interpolation algorithm (7). In addition to intracranial pressure values, we also extracted baseline data, first-day ICU variables, and mortality data for the study cohort from the MIMIC-IV v2.0 database. Baseline data included gender and admission age, while mortality data comprised ICU death, hospital stay death, and all-cause 30-day death statuses, determined based on death time records, ICU admission and discharge times, and hospital admission and discharge times. First-day ICU variables were grouped into six categories: vital signs, neurological examinations, ICU scoring, lab tests, and ventilation. For multi-measured items such as heart rate and systolic blood pressure, the mean, minimum (min), and maximum (max) values were used in accordance with clinical practice. For instance, the minimum GCS score on the first ICU Day was employed for disease severity grading. We also extracted first-day ICU input and urine output data, calculating the fluid balance.

### ICP pattern recognition and characterization

In this study, ICP pattern recognition was achieved using available time-series clustering procedures with the R package TSrepr (version 1.1.0) (7). First, the matrix of ICP values at each time point (from 1 to 24 h) for the entire study cohort was normalized using the z-score method. Next, the K-medoids clustering method was applied to the normalized ICP matrix, testing a range of cluster numbers (2–7). The optimal cluster number was determined by the Davies–Bouldin index. We employed the software X-tile (version 3.6.1) to identify the best cut-points for ICP_mean_ and ICP_variance_ (8).

### Statistical analysis

The statistical analysis were performed in R (version 4.2.1). Descriptive statistics generarted using R package tableone (version 0.13.2) (9), presenting continuous variables (e.g., WBC) as mean±SD and categorical variables (e.g., gender) as case numbers with percentages. The Kolmogorov–Smirnov normality test to guide parametric test selection (t-test/ANOVA vs. alternatives); and the treatment effect of mean- and variance-based intracranial pressure (ICP) management were analyzed using EmpowerStats (version 5.0) and the R package MatchIt (version 4.3.4) in real-world treatment effect analysis (10). The outcome variables were death during the ICU stay and death within 30 days of admission. Treatment variables were set as ICP_mean_ and ICP_variance_, with groups determined by X-tile calculated cut-points. Baseline covariates included age, gender, minimum Glasgow Coma Scale (GCS_min_), Acute Physiology Score III (APS III), Oxford Acute Severity of Illness Score (OASIS), and Sequential Organ Failure Assessment (SOFA) scores. Linear regression model treatment effects (ICP_mean_ and ICP_variance_) adjusted for baseline covariates. Confounders (*p* < 0.1 or *β* change > 0.1) informed generalized additive model-derived propensity scores (11). Stabilized inverse probability of treatment weighting with propensity scores to estimate the average treatment effect for the treated, the average treatment effect for the untreated, and the overall average treatment effect. The statistical significance threshold was set at *p* < 0.05, and the false discovery rate was controlled using the Benjamini-Hochberg procedure.

## Results

### Study cohort characteristics

After reviewing 1,143 subarachnoid hemorrhage (SAH) records from the MIMIC-IV v2.0 database, we included 176 unique records (15.4%) in the SAH study cohort. Table 1 summarizes the baseline clinical characteristics at ICU admission and during the first ICU Day. The mean age of the study cohort was 58.8 years, with a standard deviation of 14.5 years. The cohort consisted of a higher proportion of female patients (58.0%). Although the original SAH grading information was unavailable, the minimum Glasgow Coma Scale (GCS) during the first ICU Day indicated that 67 patients (38.1%) were classified as Hunt and Hess/World Federation of Neurosurgical Societies (WFNS) Grade IV, while 74 patients (42.0%) were classified as Grade V. Additional details about the MIMIC-IV database used in this study cohort can be found in Supplementary material 1.

**Table 1:** Characteristics of the SAH study cohort.

Variables	First-day normalization method	Whole cohort (*n* = 176)	Group 1 (*n* = 22): ICP_mean_ > 14 mmHg	Group 2 (*n* = 154): ICP_mean_ < = 14 mmHg	P_Value_	FDR_significance_
Basic characteristics						
Gender: Female, *n*	–	102 (58.0%)	12 (54.5%)	90 (58.4%)	0.729	insig
Gender: Male, *n*	–	74 (42.0%)	10 (45.5%)	64 (41.6%)		
Age, yrs	–	58.8 ± 14.5	52.7 ± 14.3	59.6 ± 14.4	0.036	insig
Mortalities						
ICU deaths, *n*	–	39 (22.2%)	13 (59.1%)	26 (16.9%)	<0.001	sig
Deaths during hospital admission, *n*	–	40 (22.7%)	13 (59.1%)	27 (17.5%)	<0.001	sig
Deaths within 30 days of admission, *n*	–	41 (23.3%)	13 (59.1%)	28 (18.2%)	<0.001	sig
All deaths, *n*	–	57 (32.4%)	14 (63.6%)	43 (27.9%)	<0.001	sig
ICU scores						
APS III, points	–	52.8 ± 22.6	53.0 ± 23.7	52.8 ± 22.5	0.971	insig
OASIS, points	–	37.6 ± 7.7	36.3 ± 8.6	37.8 ± 7.6	0.413	insig
SOFA, points	–	5.2 ± 2.7	5.0 ± 2.8	5.3 ± 2.7	0.671	insig
Vital signs						
Heart rate, BPM	Mean	80.3 ± 11.7	80.2 ± 13.9	80.3 ± 11.4	0.976	insig
Systolic blood pressure, mmHg	Mean	125.7 ± 11.7	128.6 ± 10.9	125.3 ± 11.8	0.227	insig
Respiratory rate, breaths/min	Mean	18.8 ± 3.1	19.3 ± 3.6	18.7 ± 3.0	0.408	insig
Body temperature, °C	Mean	37.2 ± 0.5	37.0 ± 0.8	37.2 ± 0.4	0.183	insig
Neurological examinations						
GCS_total_: 15	Min	11 (6.2%)	5 (22.7%)	6 (3.9%)	0.004	sig
GCS_total_: 13–14	Min	24 (13.6%)	1 (4.5%)	23 (14.9%)		
GCS_total_: 7–12	Min	67 (38.1%)	6 (27.3%)	61 (39.6%)		
GCS_total_: 3–6	Min	74 (42.0%)	10 (45.5%)	64 (41.6%)		
Lab tests						
Hemoglobin, g/dl	Min	11.8 ± 1.9	11.6 ± 2.1	11.8 ± 1.8	0.744	insig
Hemoglobin, g/dl	Max	13.4 ± 1.8	13.4 ± 1.7	13.4 ± 1.8	0.912	insig
WBC count, × 10^9^/L	Min	12.3 ± 4.4	11.6 ± 4.0	12.4 ± 4.4	0.459	insig
WBC count, × 10^9^/L	Max	16.4 ± 6.2	17.9 ± 7.1	16.2 ± 6.0	0.239	insig
Platelets, × 10^9^/L	Min	208.5 ± 68.6	196.6 ± 56.7	210.2 ± 70.1	0.388	insig
Platelets, × 10^9^/L	Max	244.1 ± 73.2	238.3 ± 58.9	244.9 ± 75.2	0.691	insig
Glucose, mg/dL	Min	134.7 ± 32.0	137.0 ± 39.1	134.4 ± 31.0	0.715	insig
Glucose, mg/dL	Max	181.9 ± 62.0	197.5 ± 76.3	179.6 ± 59.6	0.208	insig
Lactate, mmol/L	Max	2.4 ± 1.6	2.5 ± 2.5	2.3 ± 1.5	0.711	insig
Creatinine, mg/dL	Min	0.8 ± 0.5	0.6 ± 0.2	0.8 ± 0.5	0.159	insig
Creatinine, mg/dL	Max	0.9 ± 0.6	0.8 ± 0.2	0.9 ± 0.6	0.255	insig
Na^+^, mmol/L	Min	138.2 ± 3.7	138.0 ± 3.5	138.2 ± 3.8	0.812	insig
Na^+^, mmol/L	Max	142.8 ± 5.7	144.5 ± 4.8	142.6 ± 5.7	0.125	insig
K^+^, mmol/L	Min	3.5 ± 0.4	3.5 ± 0.4	3.6 ± 0.4	0.817	insig
K^+^, mmol/L	Max	4.2 ± 0.7	4.3 ± 0.7	4.2 ± 0.7	0.499	insig
Ventilation						
P_CO2_, mmHg	Min	33.7 ± 6.1	35.1 ± 6.1	33.5 ± 6.1	0.241	insig

### First ICU day ICP pattern

**Figure 2:**
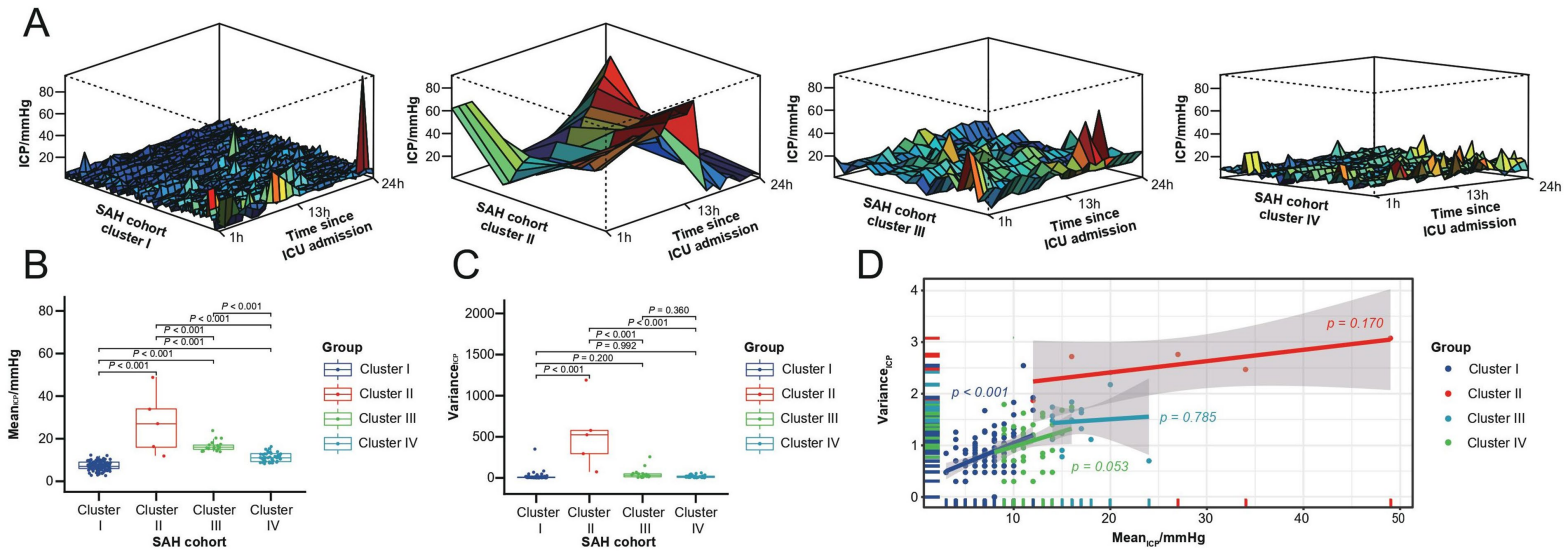
First ICU Day ICP patterns for the SAH study cohort. **(A)** Four ICP patterns identified through time series clustering in the SAH cohort, with 24-h ICP distributions for clusters I to IV shown from left to right. **(B)** Box plot of ICP_mean_ across the four clusters. **(C)** Box plot of ICP_variance_ within the four clusters. **(D)** Test line regression plot illustrating the distribution of ICP_mean_ and ICP_variance_ across the four clusters.

The time-series clustering method was employed to analyze hourly ICP data from the first ICU Day in order to identify SAH-related ICP patterns. After addressing missing data through imputation, normalizing the dataset, and selecting the optimal number of clusters, we identified four distinct ICP patterns (clusters) within our cohort (Figure 2A). Clusters II (five patients, 2.8%) and III (seventeen patients, 9.7%) exhibited notably higher ICP distributions compared to clusters I (one hundred and nine patients, 61.9%) and IV (forty-five patients, 25.6%). This difference was statistically significant upon intra-cluster comparison (*p* < 0.001, Figure 2B). Additionally, cluster II displayed a tendency for dramatic ICP level fluctuations. An intra-cluster comparison revealed that cluster II had a significantly higher variance compared to the other clusters (*p* < 0.001, Figure 2C). Taking both ICP_mean_ and ICP_variance_ into account (Figure 2D), clusters I and IV shared similarities, with an average ICP_mean_ of 9 mmHg. Clusters II and III exhibited higher average ICP_mean_ values of 19 mmHg, with cluster II also demonstrating higher ICP_variance_. Among the four clusters, a statistically significant correlation between ICP_mean_ and ICP_variance_ was only observed in cluster I.

### Correlation between ICP and SAH mortality

**Figure 3:**
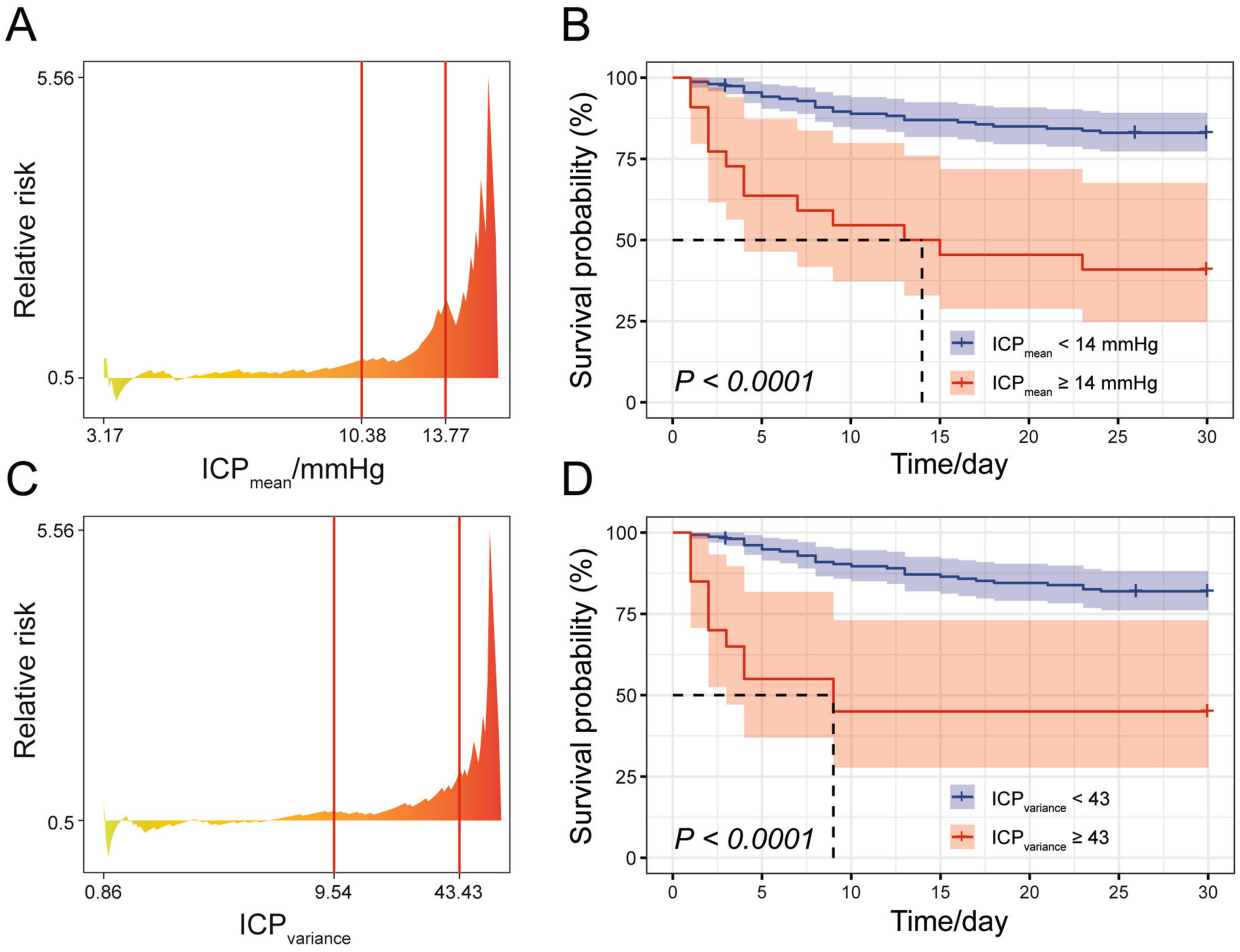
Cut-off point analysis for ICP_mean_ and ICP_variance_. **(A)** Relative risk plot for mean ICP, with X-tile recommended optimal cut-off points indicated by red lines. **(B)** Survival curve for the SAH study cohort during ICU stay, with patients divided into two groups based on the rounded ICP_mean_ cut-off point yielding the smallest log-rank *p*-value. **(C)** Relative risk plot for ICP_variance_, with X-tile recommended optimal cut-off points indicated by red lines. **(D)** Survival curve for the SAH study cohort during ICU stay, with patients divided into two groups based on the rounded ICP_variance_ cut-off point yielding the smallest log-rank *p*-value.

We investigated the clinical implications of the ICP patterns characterized by distinct ICP_mean_ and ICP_variance_ preferences by examining their correlation with SAH mortality. First, the optimal cut-off points for ICP_mean_ and ICP_variance_ were determined using X-tile software based on the survival data of the SAH cohort during their ICU stay (Figures 3A,C). When divided by the optimal cut-off points for ICP_mean_ (14 mmHg) and ICP_variance_ (43), the SAH cohort exhibited significantly different survival outcomes between groups (*p* < 0.001, Figures 3B,D).

To assess whether ICP_mean_ and ICP_variance_ could serve as management targets in SAH treatment, potentially improving survival outcomes, we estimated the treatment effect using the ICP_mean_ and ICP_variance_ groups as treatment factors. As the X-tile recommended cut-off points for ICP_mean_ (14 mmHg) and ICP_variance_ (43) resulted in imbalanced group sizes, with the larger group comprising over 80% of the SAH cohort, we employed propensity score matching to balance the distribution of potential covariates, including age, gender, GCS_min_, APS III, OASIS, and SOFA scores. The significance of the average treatment effect was only observed in ICP_mean_ -based management (Table 2). The clinical characteristics of the two ICP_mean_ groups (> 14 mmHg and < 14 mmHg) are summarized in Table 1. After false discovery rate correction, SAH mortality and GCS_min_ were the only significant variables between groups. In the multivariate analysis, ICP_mean_ emerged as the most prominent independent predictor of ICU mortality, with the highest odds ratio (OR = 22.525, 95% CI: 4.887–103.833) compared to other variables (Table 3).

**Table 2:** Treatment effect estimation for ICP management based on ICPmean and ICPvariance in the SAH study cohort.

Variables	β (95%CI)	*p*-value
ICP*_mean_*		
Average treatment effect for treated	5.5 (1.9, 15.9)	0.002
Average treatment effect	3.3 (1.1, 9.7)	0.032
ICP*_variance_*		
Average treatment effect for treated	4.2 (1.4, 12.7)	0.011
Average treatment effect	1.7 (0.5, 5.5)	0.395

**Table 3:** Logistic regression results for ICU mortality in SAH patients.

Variables	Odds Ratio (95%CI)	*p*-value
Age, yrs	1.058 (1.015–1.103)	0.007
Glucose_min_, mg/dL	1.018 (1.002–1.035)	0.031
PCO_2min_, mmHg	0.889 (0.802–0.985)	0.025
ICP*_mean_*	22.525 (4.887–103.833)	< 0.001

**Figure 4:**
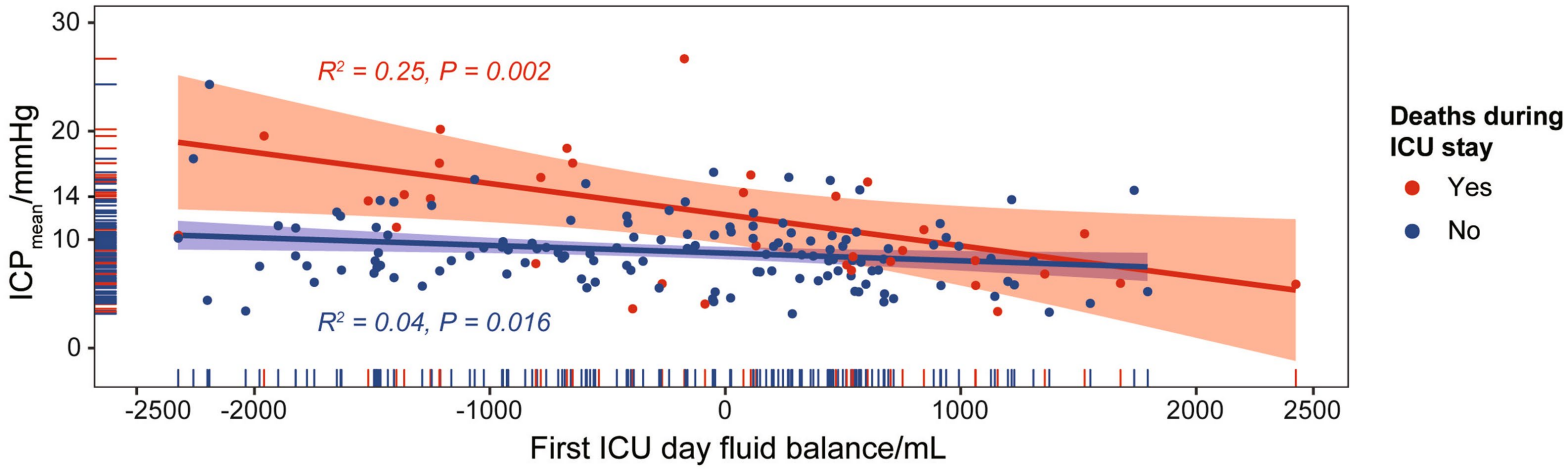
Correlation between first ICU Day fluid balance and ICP_mean_.

Fluid balance could serve as an indicator of the efficacy of ICP-targeted therapy. We discovered a significant correlation between first ICU Day fluid balance and ICP_mean_ for SAH cases with or without death during ICU stay (*p* = 0.002 and *p* = 0.016, respectively). SAH cases with ICU death had a higher *R*^2^ value than those without, suggesting that ICP-targeted therapy was less effective in patients with a higher mortality risk (Figure 4). Furthermore, an increase in SAH mortality cases was observed above the ICP_mean_ cut-off point (14 mmHg).

## Discussion

In this study, we identified four subgroups of subarachnoid hemorrhage (SAH) patients with the distinct first-day mean ICP values using continuous invasive ICP monitoring data. Our findings revealed that maintaining ICP below 14 mmHg on the first day after admission, as recommended by the X-tile, is associated with better in-hospital mortality outcomes and higher odds ratios compared to other clinical features. Notably, higher ICP values were linked to lower GCS scores at admission, emphasizing the importance of targeted ICP management for improved SAH prognosis.

SAH carries a high risk of disability and death, accounting for 19% of 28-day and 16% of 5-year mortality rates in Chinese ischemic stroke patients (12). ICP management is crucial in SAH treatment due to its connection with mortality and disease pathogenesis (13, 14). Common SAH complications, such as global cerebral edema, acute hydrocephalus, and impaired cerebral blood flow autoregulation, directly affect ICP levels (15). Clinicians require real-time ICP monitoring to maintain the ICP-mean arterial pressure gap-dependent cerebral perfusion. However, the invasive nature of ICP monitoring has limited its application in SAH management. Interventional ICP thresholds largely rely on evidence from traumatic brain injury (TBI) studies, despite the different pathogenesis and pathophysiology between SAH and TBI. Various ICP thresholds have been recommended, including 20–30 mmHg, 20–25 mmHg, 20 mmHg, and most recently, 22 mmHg (4, 16–18). While ICP monitoring is crucial for aggressive treatment and improved survival in SAH patients, the use of TBI-derived thresholds may introduce limitations and adverse effects. After SAH, strict monitoring of ICP control methods, such as direct drainage, hypertonic saline, and mannitol, is necessary to avoid aggravating the condition. Increased ICP acts as a natural protection mechanism against SAH rebleeding, making it dangerous to control ICP too low (19). This limitation of therapeutic intensity depends on establishing specific ICP targets. Therefore, this study reviewed first-day ICP changes in SAH patients within the MIMIC-IV database, aiming to find the optimal ICP threshold and its relationship with patient prognosis to provide clinical evidence-based value for SAH ICP management.

We employed an innovative unsupervised time-series clustering approach on hourly intracranial pressure (ICP) data of 176 SAH patients during their first day in the intensive care unit (ICU). The identified four distinct ICP patterns, with the second and third clusters exhibiting significantly higher average ICP values and greater fluctuations (variance) compared to the first and fourth clusters. This finding highlights the clinical relevance of continuous ICP monitoring and intervention during the initial 24 h of SAH patients’ ICU stay, as it facilitates timely feedback to avoid overtreatment and complications while providing valuable insights into patients’ prognosis. The threshold for ICP intervention in SAH patients on the first day, calculated using X-tile software, is 14 mmHg, which is consistent with recent studies (20). Patients with ICP higher than 14 mmHg warrant close attention and may require active clinical intervention. However, the ideal ICP value for initiating dedicated therapy remains uncertain, and the invasive nature of ICP monitoring may lead to selection bias. We hypothesize that ICP values change dynamically over the course of the disease, necessitating further investigation. On the first day after SAH, cerebral edema has not yet peaked; thus, the level of ICP on the first day indicates the severity of brain damage in patients at the onset of the disease, which may be associated with patient prognosis. Our research demonstrated that patients undergoing first-day ICP monitoring and maintaining ICP below 14 mmHg had better prognoses, whereas patients with ICP above 14 mmHg experienced higher mortality rates. The findings of this study provide new data-driven insights into setting ICP target values for SAH patients. In related literature, our results align closely with the ICP threshold associated with poor long-term neurological outcomes, as found in one of the two cohorts studied by Carra et al. (21). This, in turn, leads to a discrepancy with another cohort of their study. However, clinical practice demands decisiveness, expecting us to be “either cold or hot” rather than “neither cold nor hot.” To accomplish this, it would be necessary to integrate information from concurrent treatments and other interventions for a more comprehensive and clear analysis in the future.

Management and treatment principles for elevated ICP in SAH patients include head elevation at 30°, mild sedation, targeted temperature management, and osmotic therapies such as hypertonic saline and mannitol, among others (22–24). Our study also analyzed fluid therapy strategies and found no significant difference in fluid balance between patients who survived and those who died in the ICU. We discovered a weak but statistically significant association between ICP in SAH patients and fluid intake. For SAH patients with ICP higher than 14 mmHg, increasing the intensity of dehydration measures did not significantly reduce mortality. However, additional research is required to determine how to adjust fluid volume according to the first-day ICP values, especially for patients with an “extremely” high ICP level. Our clinical findings demonstrating a significant association between elevated ICP and poor functional outcomes following SAH align with a growing body of experimental evidence elucidating the underlying cellular and molecular mechanisms of ICP-mediated brain injury. Beyond hematoma-derived toxins, ICP elevation itself contributes to SAH-induced neuroinflammation. Solár et al. (25) showed that artificially increased ICP triggers macrophage accumulation and proliferation in the choroid plexus, while their later work identified TLR9 as a key mediator of ICP-driven inflammation, promoting cytokine release and monocyte recruitment via mechanosensitive pathways (26). These findings implicate ICP control as a potential strategy to interrupt this cascade. Our findings underscore the importance of continuous ICP monitoring and intervention during the first day of SAH patients’ ICU stay to improve prognosis and inform evidence-based fluid therapy strategies.

This study presents several limitations that should be considered. Firstly, the patient classification could be improved, since the Hunt-Hess grade for SAH patients was not available. GCS values were used as a substitute, but a more accurate method would be beneficial for proper stratification. Secondly, the limited sample size may introduce bias in the statistical analysis, and multiple factors, such as hemodynamic instability and clinical interventions, could affect the first day’s highest ICP value (24, 25). An expanded cohort is important for a comprehensive analysis. Thirdly, various factors, including the underlying etiologies of SAH and complications, could influence the 30-day mortality rate, with the proportion of SAH cases attributable to aneurysm rupture, including detailed aneurysm characteristics and treatment modalities, as well as relevant data on hydrocephalus, vasospasm, and blood pressure not provided. Fourthly, additional information on ICP recording methods would be necessary, as previous reports have shown the possibility of maintaining open intraventricular catheters for continuous drainage, which may lead to underestimated ICP elevations. Additionally, the limitations of the study include the unavailability of SAH source localization (e.g., aneurysm location) and treatment interventions (e.g., clipping/coiling) due to the MIMIC-IV anonymization scheme. This precludes an analysis of the relationship between the causes of hemorrhage or how acute treatments affect ICP outcomes, potentially introducing unmeasurable confounding factors. Future research should prospectively record these variables to improve ICP management strategies. Furthermore, it is important to note that ICP changes in SAH patients are dynamic. In addition to the first-day cutoff value, the value and trends of ICP and waveform changes throughout the disease course are also worth further investigation for their potential impact on prognostic assessment. Addressing these limitations calls for additional randomized controlled studies to assess the effects of the first-day ICP monitoring and intervention on patient outcomes, thereby contributing to higher-level medical evidence.

## Conclusion

In conclusion, evaluating first-day average ICP for subarachnoid hemorrhage patient stratification can offer useful prognostic information. Suggesting clinicians ought to consider tailored interventions based on specific clinical conditions when ICP surpasses 14 mmHg and adapt fluid therapy to each SAH patient’s ICP accordingly.

## Data Availability

The original contributions presented in the study are included in the article/Supplementary material, further inquiries can be directed to the corresponding authors.
